# Ecological genetics of *Juglans nigra*: Differences in early growth patterns of natural populations

**DOI:** 10.1002/ece3.7571

**Published:** 2021-05-13

**Authors:** Lauren Onofrio, Gary Hawley, Laura P. Leites

**Affiliations:** ^1^ Department of Ecosystem Science and Management The Pennsylvania State University University Park PA USA; ^2^ Rubenstein School of Environment and the Natural Resources The University of Vermont Burlington VT USA

**Keywords:** adaptation to climate, ecological genetics, *Juglans nigra*, juvenile growth patterns, provenance tests

## Abstract

Many boreal and temperate forest tree species distributed across large geographic ranges are composed of populations adapted to the climate they inhabit. Forestry provenance studies and common gardens provide evidence of local adaptation to climate when associations between fitness traits and the populations' home climates are observed. Most studies that evaluate tree height as a fitness trait do so at a specific point in time. In this study, we elucidate differences in early growth patterns in black walnut (*Juglans nigra* L.) populations by modeling height growth from seed up to age 11. The data comprise tree height measurements between ages 2 and 11 for 52 natural populations of black walnut collected through its geographic range and planted in one or more of 3 common gardens. We use the Chapman–Richards growth model in a mixed effects framework and test whether populations differ in growth patterns by incorporating populations' home climate into the model. In addition, we evaluate differences in populations' absolute growth and relative growth based on the fitted model. Models indicated that populations from warmer climates had the highest cumulative growth through time, with differences in average tree height between populations from home climates with a mean annual temperature (MAT) of 13°C and of 7°C estimated to be as high as 80% at age 3. Populations from warmer climates were also estimated to have higher and earlier maximum absolute growth rate than populations from colder climates. In addition, populations from warm climates were predicted to have higher relative growth rates at any given tree size. Results indicate that natural selection may shape early growth patterns of populations within a tree species, suggesting that fast early growth rates are likely selected for in relatively mild environments where competition rather than tolerance to environmental stressors becomes the dominant selection pressure.

## Graphical abstract

We model early growth patterns of *Juglans nigra* populations growing in common gardens and assess whether growth patterns are associated with populations' home climate. Results indicate that populations from warmer climates have the highest cumulative growth through time and reach maximum absolute growth at an earlier age than populations from colder climates. Populations from warm climates also have higher relative growth rates at any given tree size.

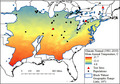

## 1 INTRODUCTION

Boreal and temperate tree species with extensive geographic ranges, such as black walnut (*Juglans nigra* L.), encounter high spatial climatic variability within their range. Within these species, survival and growth clines along climate gradients are common, resulting in a gradient of populations adapted to a segment of the species' climate range. Notably, natural populations synchronize their annual growth cycle with the frost‐free period of their respective localities (Bennie et al., 2010; Howe et al., 2003; Morgenstern, 1996). When grown in common gardens, populations exhibit differences in phenology that result in differences in the length of the growth period. Populations from colder climates, adapted to shorter frost‐free periods, generally exhibit shorter growing seasons and are thus smaller. A shorter growing season in these environments confers them frost‐hardiness and thus better cold tolerance. Conversely, populations from warmer climates, adapted to longer frost‐free periods, are taller but may be more susceptible to late spring or early autumn frosts making them less cold tolerant (Aitken & Bemmels, 2016; Campbell & Sorensen, 1978; Howe et al., 2003; Rehfeldt et al., 2004, 2018).

Genetic differences along climatic gradients in cumulative growth at a given age have been well documented for populations in many tree species (e.g., Aitken & Bemmels, 2016; Leites et al., 2019; Rehfeldt, 1988, 1989, 1990, 1991; Rehfeldt et al., 2014; Sáenz‐Romero et al., 2017; St Clair et al., 2005; Thomson & Parker, 2008). However, few studies have modeled or described the early growth patterns that lead to those differences. For many tree species, early growth patterns are crucial in determining the likelihood of tree survival (Petit & Hampe, 2006). In a regenerating forest, trees must compete not only with other tree seedlings but with herbaceous plants and woody shrubs; therefore, rapid juvenile growth is critical in early stages of forest regeneration (Petit & Hampe, 2006). In addition, when trees arrive with a competitive advantage to the stem exclusion stage (when canopy closure occurs and density‐dependent mortality begins), they are more likely to survive subsequent competition and to occupy a dominant or co‐dominant position in the new canopy (Oliver & Larson, 1996). Therefore, the taller a tree by the stem exclusion stage, the higher the likelihood that it will outcompete neighboring individuals (Oliver & Larson, 1996; Weiner, 1990).

Early growth rates (defined as the first 10 years) are more important in climatically mild sites where competition with other vegetation is high and stem exclusion stage is reached earlier. Populations from climatically milder sites grow more per year due to their longer growing seasons. However, it is also likely that natural selection may favor faster intrinsic early growth rates at these sites. Previous studies indicate that growth rate in plants is related to environmental factors that influence productivity and competition dynamics (Dmitriew, 2011; Rose et al., 2009; Rosielle & Hamblin, 1981). Slow growth rates are more common in climatically stressful environments, while fast growth rates are more common in resource‐rich or climatically mild environments where competition is likely to be higher (Chapin et al., 1993; Kimball et al., 2013; Weis et al., 2000). The combination of longer growing seasons with potentially higher growth rates in populations from milder climates could be reflected in growth patterns characterized by rapid growth early on and reaching the inflection point on the sigmoidal curve that characterizes tree growth over time earlier than populations from colder environments.

Modeling differences in early growth patterns that arise from differences in home climate is critical to better understand tree species strategies to adapt to climate and their potential responses to climate change. Modeling the growth of local and nonlocal populations is also critical to plan assisted migration strategies (Aitken & Bemmels, 2016). From an applied standpoint, modeling differences in early growth patterns within a species can aid in the identification of optimal seed sources for a given site and lead to the improvement of forest growth models that currently model growth at the species level as the most basic unit (e.g., Crookston & Dixon, 2005). Despite its importance, we know of no study that models differences in early growth patterns of different natural populations within an ecological genetics context.

In this study, we model early growth patterns of natural black walnut populations grown in common gardens and test for differences shaped by home climate. Following classical growth analyses (e.g., Paine et al., 2012; Pienaar & Turnbull, 1973; Pommerening & Muszta, 2016), we model cumulative growth, absolute growth rate, and relative growth rate of 52 natural populations between ages 2 and 11 grown from seed and test whether home climate is a significant predictor of growth patterns. We use data from provenance trials (common gardens), where differences among populations can be attributed to genetic differences (Davis et al., 2005; Etterson et al., 2016; Kremer et al., 2014), and where clinal association between growth traits and the natural populations' home climate can be interpreted as evidence for genetic adaptation to climate (Aitken et al., 2008; Alberto et al., 2013; Campbell & Sorensen, 1978; Rehfeldt, 1984).

We focus on black walnut as a case study for two reasons. First, prior studies have found that black walnut exhibits significant patterns of genetic variation in height growth along climatic gradients (Leites et al., 2019) and strong differentiation among populations in several other fitness traits (e.g., Bey, 1973, 1979; Wright & Lemmien, 1972). Early growth patterns are very important for this shade‐intolerant species (Baker, 1949) that, in mixed forest stands, must reach a dominant position by stem exclusion phase to survive (Burns & Honkala, 1990). Second, fewer studies have focused on ecological genetics of broadleaf deciduous as compared to conifers, and therefore, this study also aims to fill that knowledge gap.

## 2 METHODS

**FIGURE 1:**
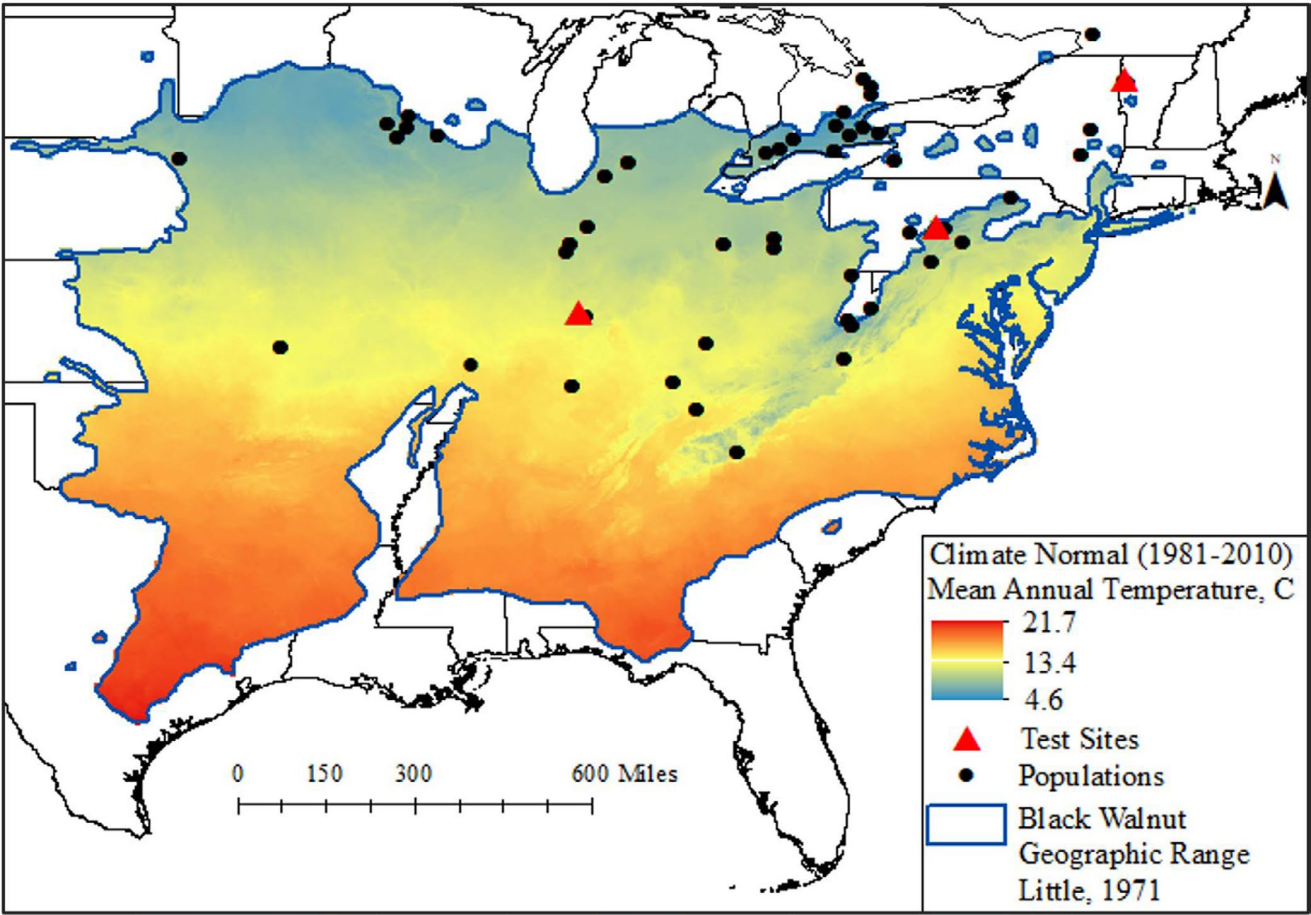
Distribution of evaluated populations and test sites in relation to the mean annual temperature of the species range

We used published and unpublished data from two provenance test (common gardens) series, one established in 1967 (Bey, 1973; Bey & Williams, 1974) and another established in 1980 (Waite et al., 1988). These experimental series comprised three test sites located in Indiana, Pennsylvania, and Vermont, USA, and evaluated a total of 92 natural populations from the black walnut range (Figure 1; Table 1). The populations tested spanned a latitudinal range of 35.28–45.50 degrees and a longitudinal range of −96.40 to −73.17 degrees. Each test site followed a randomized complete block design (6 blocks in Indiana and Vermont, and 5 in Pennsylvania), with four‐tree row plots. Each population was, then, represented by 4 trees per block for a total of 20–24 trees depending on the number of blocks in the study. Seedlings were 1 year old at planting and were planted 3.7 m apart in Indiana, 3 m apart in Pennsylvania, and 2.5 m apart in Vermont. At each site, total tree height was recorded at several ages between 2 and 11 years from seed for all populations (Table 1). We used the average tree height of each population at each age and test site. Survival was 90% in Pennsylvania after 6 growing seasons and 91% in Vermont after 7 growing seasons. In Indiana, survival after 7 growing seasons was 61% due to partial flooding and root rot issues in the earlier years; however, survival was not correlated with population origin (Bey & Williams, 1974). In the Indiana test, *Alnus glutinosa* was planted around each test tree at the beginning of the third growing season. We assume these trees did not provide competition to the test trees during the measured ages of at least 7. Climate normals for mean annual temperature for the period of 1961–1990 for all populations and test sites were obtained from Rehfeldt's climate surfaces for North America at 1 km resolution (Rehfeldt, 2006; data available at http://charcoal.cnre.vt.edu/climate); this time period represents the climate prior to seed collection and thus is a good representation of the population's home climate as well as the climate during the test period. In this study, we used observations where populations were transferred to a test site within ±2°C of the population home climate to minimize the effect of transfer distance in the expression of innate growth potential for each population (Rehfeldt, 1990; Rehfeldt et al., 1999). Several studies have demonstrated the negative effect of transfer distance on growth potential, with maximum growth occurring close to home climate and decreasing as transfer distance increases in absolute terms (e.g., Carter, 1996; Rehfeldt et al., 1999; Wang et al., 2006; Leites et al., 2012). Therefore, by constraining the observations, we limit the expression of the interaction between genotype and environment (*G* × *E*), an effect that masks the responses we aim to model. The ±2°C range was chosen after examining the relationship between tree height and mean annual temperature transfer distance and reviewing other studies that model the same relationship for other species (e.g., Wang et al., 2006; Leites et al., 2012 among others). Accounting for climatic transfer distance (difference between the climate of origin and test site) was not possible due to the number of observations available and the model complexity. In total, 52 natural populations and 342 observations were used in this study (Figure 1; Table 1).

**TABLE 1:** Summary of provenance test characteristics, including establishment year, number of populations tested, test site mean annual temperature (MAT), range of MAT of the populations tested, and ages from seed measured at each test site

Site	Year established	No. of populations	Test MAT (°C)	Pop. MAT range (°C)	Ages measured (years)
Vermont	1980	29	7.1	5.4–8.9	2–7
Pennsylvania	1980	19	9.4	7.7–10.9	2–11
Indiana	1967	10	11.8	10.4–13.7	3–11

To model population nonlinear height growth with age, we utilized the Chapman–Richards (Chapman, 1961; Richards, 1959) growth function in a mixed effects framework (Equation 1). The Chapman–Richards is a sigmoid function based on the early work by Von Bertalanffy (Von Bertalanffy, 1957), and it is commonly used to model tree or forest growth as a function of age. It has three parameters that determine its shape, rate, and asymptote. Variation in the rate‐related parameter leads mainly to changes in how fast growth reaches the asymptote, while variation in the shape‐related parameter leads mainly to changes in the inflection point location. The asymptote indicates maximum growth. We present a graphical illustration of the effects of varying the function parameters in Appendix A.

To model early growth patterns with this model, we considered test site and population as random effects affecting the asymptote and age as a fixed effect. We chose to incorporate the test site as a random effect on the asymptote ($\beta_{00}$) because site productivity is a strong determinant of maximum tree height (Oliver & Larson, 1996).

To test for population differences in growth patterns related to their adaptation to climate, we added mean annual temperature of the population's home climate (MAT) as a fixed effect affecting the rate‐related $\left(\beta_{10}\right)$ and shape‐related $\left(\beta_{20}\right)$ parameters of Equation (1). That is, we fit the model with the original parameters and then a model where each of those parameters was substituted by a linear function of populations' MAT (Equation 2); $\beta_{10}$ becoming $\beta_{10}+\beta_{11}{\text{MAT}}_{i}$ and $\beta_{20}$ becoming $\beta_{20}+\beta_{21}{\text{MAT}}_{i}$.

In a common garden, observed clines in fitness traits along climate gradients are interpreted as evidence for genetic differentiation in adaptation to climate (Campbell & Sorensen, 1978; Davis et al., 2005; Etterson et al., 2016; Rehfeldt, 1984); therefore, adding MAT to the model in this way tests whether there is an association between home climate and the early growth patterns and how home climate affects those patterns. We chose MAT because of previously observed clines with tree height in black walnut (Leites et al., 2019) and in many tree species (reviewed by Aitken & Bemmels, 2016). If populations' MAT improved model fit and its parameter was statistically different from zero, it provides evidence that early growth patterns differ among populations and that such differentiation is likely driven by adaptation to climate. We did not incorporate MAT affecting the asymptote parameter to avoid overparameterization and to reflect the aforementioned relationship between maximum height and site quality.

We evaluated models where population MAT affected either the rate‐related $\left(\beta_{10}\right)$ or the shape‐related $\left(\beta_{20}\right)$, and both simultaneously (full model presented in Equation 2). To evaluate the improvement to model fit, we used Akaike's information criterion (AICc; Akaike, 1974), and to evaluate the statistical significance of the fixed effect parameters, we used a *t* test with an *α*‐level of 0.05. To further evaluate the models, we also calculate root mean square prediction error (RMSPE) by age using leave‐one‐out cross‐validation.

(1) $$Ht_{\mathrm{ijk}}=\left(\beta_{00}+u_{j}+u_{i\left(j\right)}\right)*{\left(1‐e^{\beta_{10}Age_{k}}\right)}^{\beta_{20}}+\varepsilon_{\mathrm{ijk}}$$

(2) $$Ht_{\mathrm{ijk}}=\left(\beta_{00}+u_{j}+u_{i\left(j\right)}\right)*{\left(1‐e^{\left(\beta_{10}+\beta_{11}{\text{MAT}}_{i}\right){\text{Age}}_{k}}\right)}^{\beta_{20}+\beta_{21}{\text{MAT}}_{i}}+\varepsilon_{\mathrm{ijk}}$$

where Equation (1) is the baseline model and Equation (2) is the model with MAT affecting both the rate‐related $\left(\beta_{10}\right)$ and the shape‐related $\left(\beta_{20}\right)$ parameters.$Ht_{\mathrm{ijk}}$ is the average height in dm (cumulative height) of population *i* in site *j* at age *k*.$\beta_{00},\beta_{10}+\beta_{11},\;\text{and}\;\beta_{20}$+$\beta_{21}$ are parameters for the asymptote, rate‐related, and shape‐related, respectively.${\text{MAT}}_{i}$ is the mean annual temperature of population's home climate *i*, ${\text{Age}}_{k}$ is the population age, $u_{j}$ and $u_{i\left(j\right)}$ are the random effects for site and population nested within site, and $\varepsilon_{\mathrm{ijk}}$ is the error term.

We constructed bootstrapped 95% prediction confidence intervals for the final model by using the variance–covariance matrix of the parameter estimates and resampling (*n* = 1,000) parameter estimates from the multivariate normal distribution to produce a range of predictions. We then used the 2.5 and 97.5 percentiles of the resampled predictions as the upper and lower bounds of the interval. The final model form was used to calculate absolute growth rate per year (Pommerening & Muszta, 2016, Appendix B). Relative growth rates were calculated as the ratio of the absolute growth rate to cumulative growth for a given year (Appendix B). All analyses were performed in the statistical environment R (v.3.6.2 RStudio Team 2019). To fit mixed effects models, we used the package lme4 (Bates et al., 2015).

## 3 RESULTS

**FIGURE 2:**
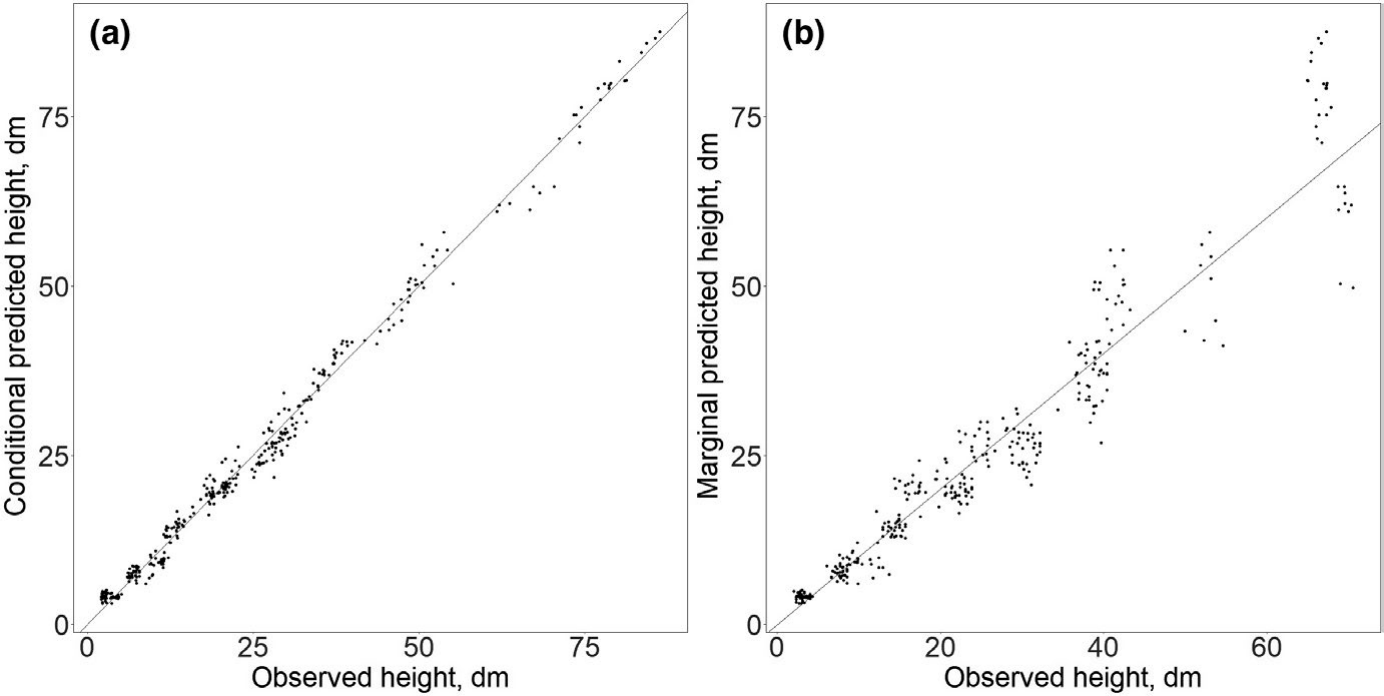
Observed versus predicted heights using the selected model, (a) conditional predicted heights (using fixed and random effects) versus observed heights, (b) marginal predicted heights (using only fixed effects) versus observed heights

The parameter estimates for the evaluated models are presented in Table 2. MAT improved model fit when added to the rate‐related parameter *β_10_* (Table 2, model 2), to the shape‐related parameter *β*_20_ (Table 2, model 3), and when added to both the shape‐related parameter *β*_20_ and the rate‐related parameter *β*_10_ (Table 2, model 4). Model 3 had the lowest AICc but it was within 2 units of the AICc for model 2, which indicates that according to this metric, both models fitted the data equally well. However, model 2 has much larger standard errors for its parameter estimates, especially for *β*_0_ (see confidence intervals in Table 2). In addition, the RMSPE values by age indicate that model 2 does not perform as well in older trees (Appendix C). For these reasons, we selected model 3 as the best model (Figure 2, additional diagnostic plots for this chosen model are presented in Appendix D).

**TABLE 2:** Parameter estimates, their approximate 95% confidence interval (C.I., *t*‐value = 2), and model AICc for models tested

Model	(1) Base model		(2) Rate‐related model		(3) Shape‐related model		(4) Rate‐ and shape‐related model	
Parameter	Estimate	C.I.	Estimate	C.I.	Estimate	C.I.	Estimate	C.I.
*β*_00_	86.8*	86.78, 86.82	90.41*	76.9, 105.9	92.24*	92.21, 92.27	92.00*	77.4, 106.6
*β*_10_	−0.225*	−0.239, −0.220	−0.13*	−0.16, −0.10	−0.21*	−0.22, −0.20	−0.19*	−0.26, −0.13
*β*_11_			−0.0093*	−0.011, −0.007			−0.0015	−0.01, 0.01
*β*_20_	3.18*	3.16, 3.20	3.11*	3.09, 3.13	4.22*	4.19, 4.25	4.04*	3.12, 4.96
*β*_21_					−0.13*	−0.14, −0.12	−0.11*	−0.21, −0.01
*σ*_Site_	12.44		11.83		11.71		11.73	
*σ*_Pop(Site)_	5.95		8.71		7.39		7.60	
*σ*_Residual_	2.27		1.92		1.98		1.97	
AICc	1,666		1,607		1,605		1,617	

Note

Parameter significance at *α* = 0.05 is indicated by *.

**FIGURE 3:**
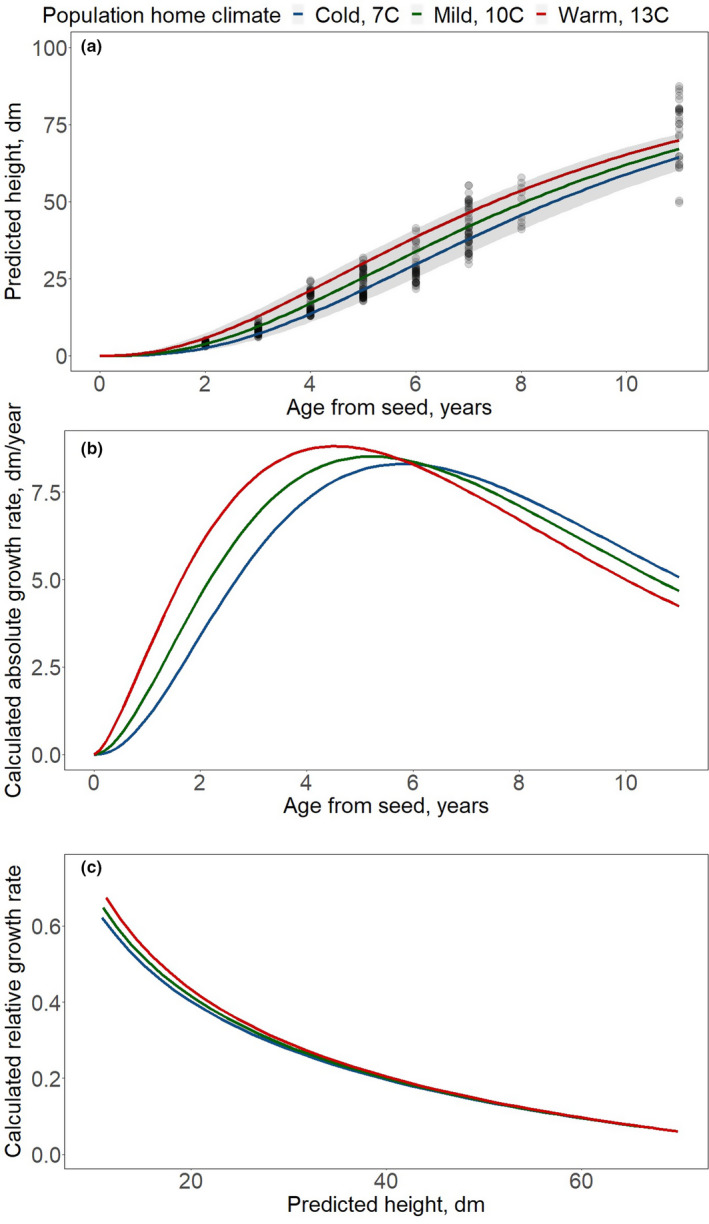
(a) Predicted population tree height using the selected model for three hypothetical populations originating in cold (7°C MAT), mild (10°C MAT), and warm (13°C MAT) climates. The 95% prediction confidence interval is represented by the gray band. Gray circles represent observations. (b) Calculated absolute growth and (c) calculated relative growth rates for the same three hypothetical populations

Model 3 indicates that population MAT has a negative relationship with the shape‐related parameter; that is, for populations from climates with higher MAT, the shape‐related parameter is smaller, leading to populations reaching the inflection point of the growth curve earlier. To illustrate the effect of the population MAT on the growth patterns, we graphed the cumulative growth model predictions and the calculated absolute growth and relative growth rates for three hypothetical populations (Figure 3). The MATs of those populations were chosen not to illustrate the maximum genetic variation within the species, but to illustrate the effect of MAT within the range of observed data (Figure 1). The differences in early growth patterns between populations from the coldest and warmest areas of the species distribution are likely more striking than the ones presented here.

### 3.1 Cumulative growth

Compared to populations from cold climates, populations from warm climates exhibit faster growth from an early age, these differences decrease through time. At age 3, populations with a MAT of 13°C are predicted to be 80% taller than populations with a MAT of 7°C, by age 6 they are predicted to be 30% taller, and by age 11 they are predicted to be 8.6% taller (Table 3 and Figure 3a).

**TABLE 3:** Predicted growth responses for hypothetical populations originating in cold (7°C) and warm climates (13°C)

Predicted response	Age, years	Hypothetical home climate		
		7°C	13°C	Percent change (%)
Cumulative height, dm	3	7.1	12.8	80.3
	6	29.7	38.5	29.8
	11	64.3	69.9	8.6
Absolute growth rate, dm/year	3	5.7	7.9	38.9
	6	8.3	8.3	0.0
	11	5.1	4.2	−16.3
Age of maximum absolute growth rate, years		5.81	4.54	

	Cumulative height, dm		
Relative growth rate, percent (in decimal format) of size/year	10	0.65	0.73
	30	0.28	0.29
	60	0.09	0.10

Note

For cumulative height and absolute growth rate, percent change was calculated using the value at MAT 7°C as the baseline ((the value at MAT of 13°C − the value at MAT of 7°C)/the value at MAT of 7°C).

### 3.2 Absolute growth rates

Populations from warm climates have higher maximum absolute growth rate and reach it earlier in age compared to populations from cold climates. Populations with a MAT of 13°C are predicted to reach their maximum absolute growth rate of 8.8 dm/year by age 4.5, whereas populations with a MAT of 7°C will not reach their maximum AGR of 8.3 dm/year until age 5.8. The age of maximum absolute growth rate decreases by 0.2 years for each additional degree Celsius in MAT. The maximum absolute growth rate decreases by 0.1 dm/year for each decreased °C in MAT (Table 3 and Figure 3b). After age 6, the higher absolute growth rate of populations from cold climates is the result of a smaller tree size.

### 3.3 Relative growth rates

Populations from warm climates had higher relative growth rates at any given tree size (Table 3 and Figure 3c). Trees 10 dm tall from populations with a MAT of 7°C are predicted to grow 65% of their size while the same sized trees but from populations from a climate with a MAT of 13°C are predicted to grow 73% of their size (Table 3). Differences between populations decrease with tree height, which point to differences in the importance of larger sizes (taller trees) early on among the different populations.

### 3.4 Test effects

**FIGURE 4:**
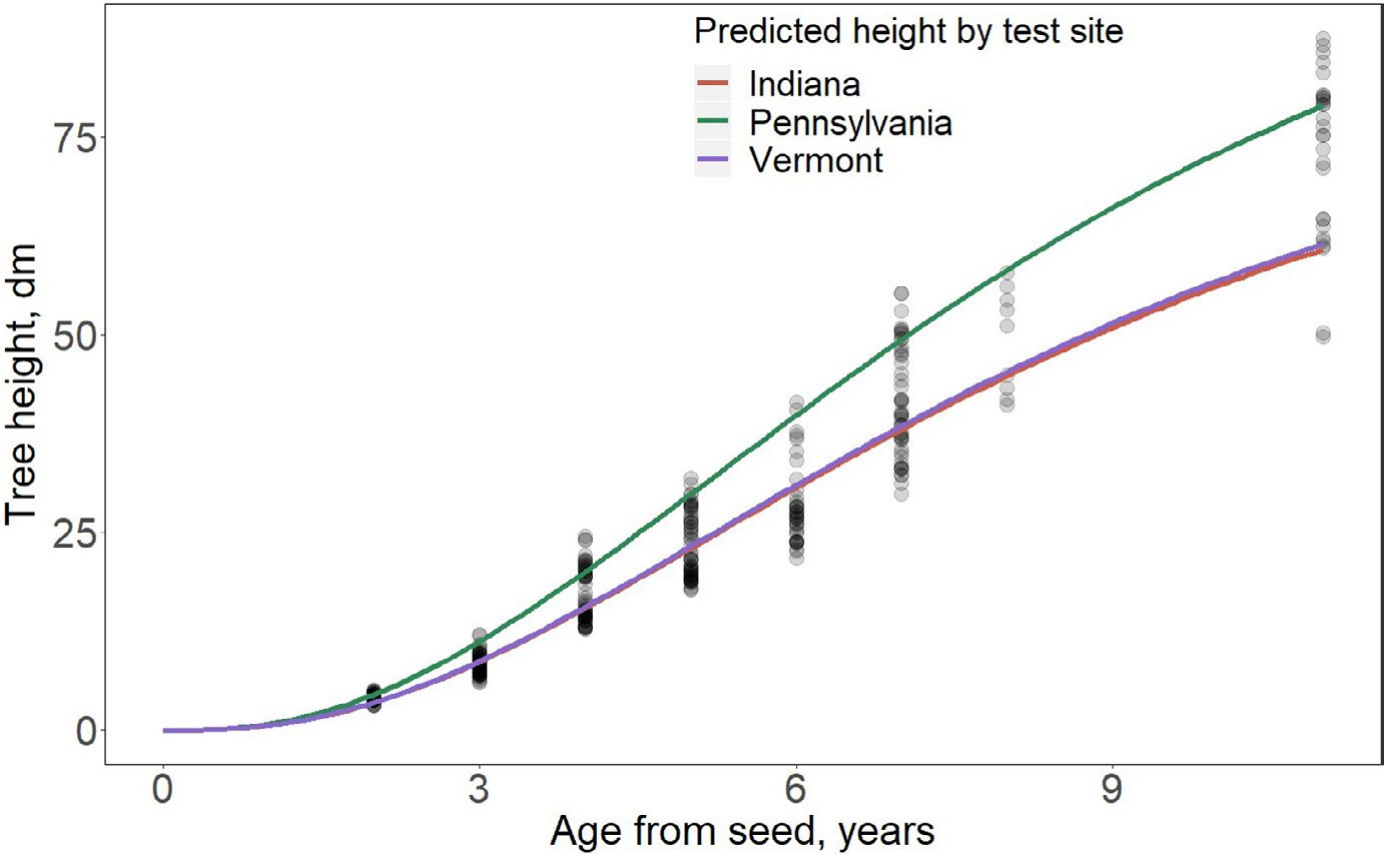
Differences in predicted height growth for each test site. Each line represents the predicted average height trajectory for each test site. Gray circles represent observations

Site effects were accounted for in the random effect and were likely driven by differences in site quality and management, not climate (Figure 4). Although the Indiana test site has the highest mean annual temperature (11.8°C), it performed similarly to the Vermont test site which has a mean annual temperature of 7.1°C, while the Pennsylvania test had the largest random effect estimate (larger asymptote) having a mean annual temperature of 9.4°C.

## 4 DISCUSSION

In this study, we modeled the early growth patterns of natural black walnut populations growing in common gardens and found evidence of genetic differentiation related to populations' climate. Our models indicated that populations with the highest cumulative growth originated in warmer home climates (as measured by MAT), while populations from colder climates were smaller. In fact, this difference was present at age 2 from seed, which is the start of the measurement period. By analyzing absolute growth curves, we also found evidence of an association between the age at which maximum absolute growth occurs and home climate (Figure 3b), which has not been reported in previous studies that focused on differences in cumulative growth at a point in time. In general, populations from warmer climates achieved maximum absolute growth earlier than those from colder climates (4.5 vs. 5.8 years), which would provide a competitive advantage early on to populations from warmer climates. By analyzing relative growth rates, we found that, at any given tree height, populations from warmer climates had faster relative growth rates. Relative or intrinsic growth rate represents the growth in proportion to the size and reflects the efficiency with which plants produce new tissue (Pommerening & Muszta, 2016). In these terms, populations from warm climates seem more efficient than populations from colder climates, at least until attaining a certain stature.

Part of the modeled differences in early growth patterns can be explained by the well‐documented differences in phenology among populations of several tree species. These phenological differences, such as differences in the timing of bud break and bud set, translate partly into differences in growing season lengths and thus differences in cumulative growth (reviewed by Aitken et al., 2008). In black walnut, early studies documented earlier bud set and leaf senescence in northern populations (Schmitt & Carter, 1987; Waite et al., 1988), and either no differences in bud burst (Waite et al., 1988) or southern populations leafing out earlier than northern populations (Bey, 1972; Wright & Lemmien, 1972), which suggest a longer growing season in southern (warmer climate) populations. The importance of competition during seedling and sapling stage, and differences in competition levels in climatically and ecologically disparate sites are factors that also likely play a role in these early growth patterns. After a stand replacing disturbance, the rapid height growth of seedlings in a regenerating forest is critical for seedling survival as they compete for light with other vegetation. Furthermore, the size of the sapling during the stem exclusion stage of forest development is a determinant of tree survival as density‐dependent mortality occurs (Oliver & Larson, 1996). Therefore, fast early height growth rates lead to taller trees better able to capture light resources and survive competition (Petit & Hampe, 2006). Competition is higher in climatically milder sites, and stem exclusion stage is reached earlier (Oliver & Larson, 1996). In these sites, natural selection is likely driven, at least in part, by competition dynamics. The early growth patterns modeled here, where populations originating in warmer climates have higher cumulative height between ages 2 and 11 (the age range of the study), reached absolute growth earlier, and had higher relative growth rates than populations originating in colder climates hint at the role competition may have as selection pressure in warmer localities.

Conversely, it is possible that for populations adapted to cold climates, survival is more dependent on cold tolerance than overcoming competition (Hänninen, 2016). By shortening their growing seasons, cold adapted populations avoid damage due to early‐ and late‐season frosts; therefore, they are more cold tolerant but exhibit less cumulative growth (Morgenstern, 1996). Natural selection may also favor tolerance traits linked to slower growth rates in harsh/resource‐limited environments (Chapin et al., 1993; Kimball et al., 2013; Weis et al., 2000). The trade‐off between cold tolerance and growth potential has been well documented in tree species (Aitken & Bemmels, 2016; Howe et al., 2003; Leites et al., 2019; Rehfeldt, 1991) and more generally as an ecological strategy in all plant species (Grime, 1979). For trees, the association of the age of maximum absolute growth with home climate observed here provides further evidence of the trade‐off between competition and stress tolerance. We also found that population differences in cumulative, absolute, and relative growth rates diminish through time. This is likely due to the initiation of competition among trees in the common gardens as they approached age 11. This competition would obscure the expression of genetic differences and likely decrease observed differences (e.g., Foster, 1986; Franklin, 1979; Rehfeldt et al., 1991). Survival in all test sites was high and unrelated to population origin (Bey & Williams, 1974; Waite et al. 1988; Steiner unpublished data). However, in Indiana, *Alnus glutinosa* trees were planted around each test tree during the third growing season; therefore, it is possible that competition affected the last two measurements at age 7 and 11 and may explain the decrease in differences among populations through time.

The differences in early growth patterns that we report support the hypothesis that intraspecific competition may be responsible for the displacement of populations from their optimal climate to colder climates within the species geographic range (Matyas & Yeatman, 1992; Namkoong, 1969; Rehfeldt et al., ,^2004^, ^2018^). This displacement has been termed “Namkoong's suboptimality concept” (Rehfeldt et al., 2018) and refers to the observation that populations of many tree species inhabit climates slightly colder than optimal, with populations adapted to colder climates showing a larger lag between their ecological and physiological optima (Rehfeldt et al., 2018). That is, there is a difference between the population's ecological optimum, the climate where it competitively excludes other populations, and the population's physiological optimum, where the population achieves maximum growth but is excluded by faster growing populations (Namkoong, 1969; Rehfeldt et al., 2018). The large differences in stature as well as the earlier age of maximum absolute growth for populations from warmer climates could lead to populations from colder climates being competitively excluded by those with higher growth rates. In each new generation, the balance between competitive exclusiveness and stress tolerance needs to be re‐established, as explained in Rehfeldt et al. (2004). At this point, asymmetric gene flow from the center of the distribution to the periphery interacts with competition‐driven selection generating populations that do not inhabit their physiological climate optimum. Therefore, the distribution of populations within a species range is the result of a balance between selection for competitive ability and for stress tolerance (e.g., Loehle, 1998; Howe et al., 2003; Rehfeldt, 2004; Bennie et al., 2010). Our work provides evidence that these processes may be operative in black walnut.

Finally, we used data from three test sites and included populations with MAT ≤ |2| °C of the test site MAT to reduce the effect of climate transfer distance (i.e., *G* × *E*). As a result, the tests and populations are not fully crossed (i.e., each population is not tested in each test site). This limitation presents the possibility that effects attributed to MAT could be partly confounded with the test effect. However, we believe that the confounded test effect may not be important for two reasons. First, the magnitude of the test effect on the overall growth does not correlate with the test MAT (Figure 4). If these differences were confounded with test site climate, we would expect populations to perform best at the warmest test site. Although the Indiana test site has the highest mean annual temperature (11.8°C), the site's average growth was similar to that of the coldest test site in Vermont (7.1°C). The Pennsylvania site average growth was highest with a mean annual temperature of 9.4°C. Second, diagnostic plots (Appendix D) show that the model fits all tests and populations equally well, making the confounding effect less likely to exist. If it existed, differences in residual distribution by site would likely be observed.

## 5 CONCLUSIONS

Models of black walnut early growth patterns indicate differentiation related to climate of origin. Populations originating in warmer climates grow faster in tree height than populations originating in colder climates. They also achieve maximum absolute height growth at an earlier age and have higher relative growth rates. Our results highlight the role that natural selection may play in driving differences in early height growth patterns among populations to increase adaptation to local climate. Our results also have ecological implications for climate change adaptation and practical implications for forest growth models.

## CONFLICT OF INTEREST

No conflict to declare.

## AUTHOR CONTRIBUTIONS

**Lauren Onofrio:** Conceptualization (equal); data curation (lead); formal analysis (equal); methodology (equal); writing‐original draft (equal). **Gary Hawley:** Resources (equal); writing‐review & editing (equal). **Laura P. Leites:** Conceptualization (equal); data curation (supporting); formal analysis (equal); funding acquisition (lead); methodology (equal); writing‐original draft (equal).

## Figures and Tables

**FIGURE A1:**
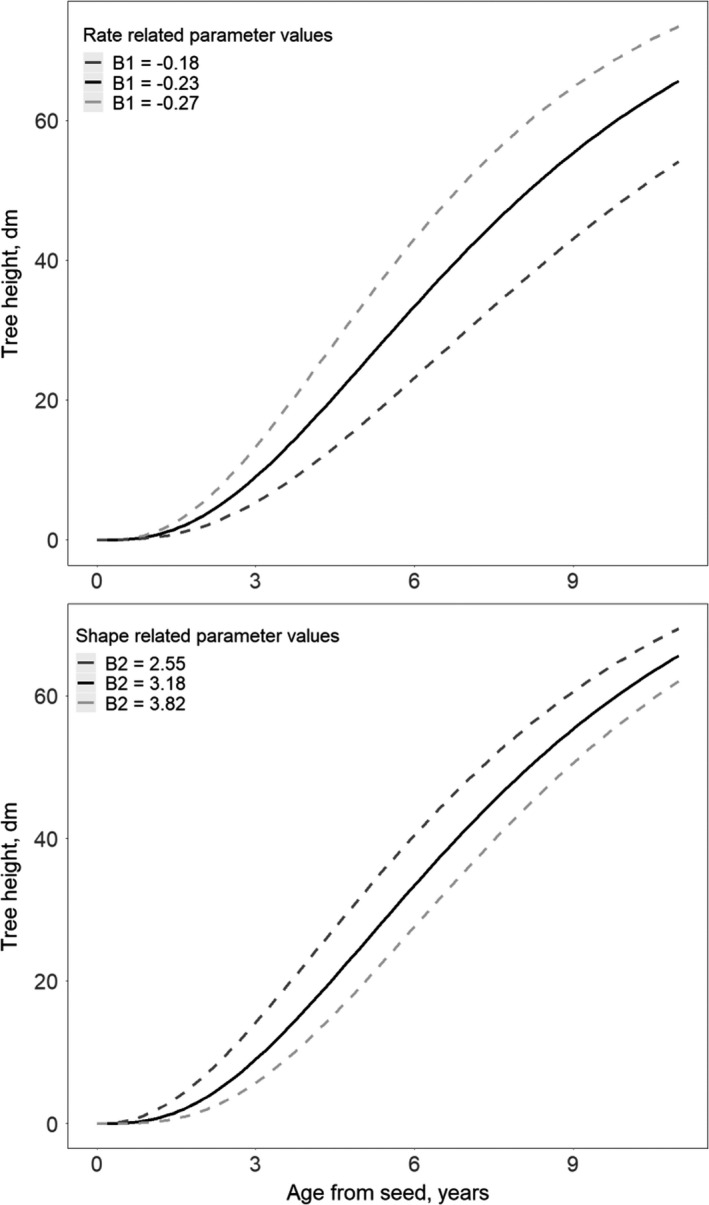
Using parameters for the base model (Table 2), the base model prediction is presented in solid line, while in (a) the rate‐related (b_1_) parameter is modified by ±20% of original value (black and gray dashed lines), and in (b) the shape‐related parameter (b_2_) is modified by ±20% of original value (black and gray dashed lines)

**FIGURE D1:**
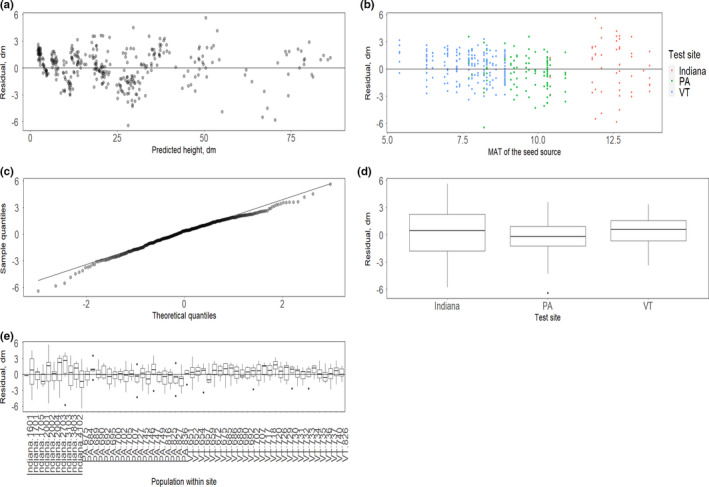
Diagnostic plots for final model, (a) residuals versus predicted height, (b) residuals versus population home climate colored by test site, (c) normal quantiles plot for the residuals, (d) residuals variation by test site, and (e) residuals variation by population within site

## Data Availability

Data used in this study are available in Dryad: Data from Ecological genetics of Juglans nigra: differences in early growth patterns of natural populations. https://doi.org/10.5061/dryad.6t1g1jwz9.

## APPENDIX A

Diagram showing the effects of varying either the shape‐related or the rate‐related parameters in the Chapman–Richards model form.

## APPENDIX B

Model forms used to calculate absolute (B1) and relative (B2) growth rates from model 3 (Table 2).

(B1) $${\text{AGR}}_{\text{Age}}=\beta_{00}*\beta_{10}*\left(\beta_{20}+\beta_{21}*\text{MAT}\right)*e^{\beta_{10}*\text{Age}}*{\left(1‐e^{\beta_{10}*\text{Age}}\right)}^{(\beta_{20}+\beta_{21}*\text{MAT})‐1}$$

(B2) $${\text{RGR}}_{\text{Age}}=\frac{{\text{AGR}}_{\text{Age}}}{{\text{Cumulative growth}}_{\text{Age}}}$$

where AGR is the absolute growth rate in dm/year, RGR is the relative growth rate in percent (expressed as decimals) per year. Cumulative growth in dm is calculated using model 3, and $\beta_{00},\beta_{10},\;\text{and}\;\beta_{20}$+$\beta_{21}$ are parameters for the asymptote, rate‐related, and shape‐related, respectively. MAT is the mean annual temperature of the population, and Age is the population age.

## APPENDIX C

Root mean square prediction error (RMSPE) by age for the evaluated models. RMSPE was calculated using leave‐one‐out cross‐validation.

**TABLE C1:** Table of RMSPE by age for each of the evaluated models

Age from seed	Rate‐related model	Shape‐related model	Rate and shape related model
2	1.25	1.29	1.29
3	1.71	1.85	1.89
4	2.98	2.98	3.03
5	3.26	3.28	3.30
6	4.9	5.17	5.12
7	6.23	6.31	6.45
8	9.10	7.37	7.20
11	13.92	13.00	13.03

## APPENDIX D

Diagnostic plots for the selected model (Table 2, Model 3).
